# Synaptonemal Complex Protein 3 Is a Prognostic Marker in Cervical Cancer

**DOI:** 10.1371/journal.pone.0098712

**Published:** 2014-06-06

**Authors:** Hanbyoul Cho, Kyung Hee Noh, Joon-Yong Chung, Mikiko Takikita, Eun Joo Chung, Bo Wook Kim, Stephen M. Hewitt, Tae Woo Kim, Jae-Hoon Kim

**Affiliations:** 1 Department of Obstetrics and Gynecology, Gangnam Severance Hospital, Yonsei University College of Medicine, Seoul, Republic of Korea; 2 Institute of Women's Life Medical Science, Yonsei University College of Medicine, Seoul, Republic of Korea; 3 Laboratory of Infection and Immunology, Graduate School of Medicine, Korea University, Ansan-Si, Gyeonggi-Do, Republic of Korea; 4 Department of Biochemistry, Korea University College of Medicine, Seoul, Republic of Korea; 5 Tissue Array Research Program, Laboratory of Pathology, Center for Cancer Research, National Cancer Institute, National Institutes of Health, Bethesda, Maryland, United States of America; 6 Radiation Oncology Branch, Center for Cancer Research, National Cancer Institute, National Institutes of Health, Bethesda, Maryland, United States of America; University of Quebec at Trois-Rivieres, Canada

## Abstract

Synaptonemal complex protein 3 (SCP3), a member of Cor1 family, is up-regulated in various cancer cells; however, its oncogenic potential and clinical significance has not yet been characterized. In the present study, we investigated the oncogenic role of SCP3 and its relationship with phosphorylated AKT (pAKT) in cervical neoplasias. The functional role of SCP3 expression was investigated by overexpression or knockdown of SCP3 in murine cell line NIH3T3 and human cervical cancer cell lines CUMC6, SiHa, CaSki, and HeLa both *in vitro* and *in vivo*. Furthermore, we examined SCP3 expression in tumor specimens from 181 cervical cancer and 400 cervical intraepithelial neoplasia (CIN) patients by immunohistochemistry and analyzed the correlation between SCP3 expression and clinicopathologic factors or survival. Overexpression of SCP3 promoted AKT-mediated tumorigenesis both *in vitro* and *in vivo*. Functional studies using NIH3T3 cells demonstrated that the C-terminal region of human SCP3 is important for AKT activation and its oncogenic potential. High expression of SCP3 was significantly associated with tumor stage (*P* = 0.002) and tumor grade (*P*<0.001), while SCP3 expression was positively associated with pAKT protein level in cervical neoplasias. Survival times for patients with cervical cancer overexpressing both SCP3 and pAKT (median, 134.0 months, *n* = 68) were significantly shorter than for patients with low expression of either SCP3 or pAKT (161.5 months, n = 108) as determined by multivariate analysis (*P* = 0.020). Our findings suggest that SCP3 plays an important role in the progression of cervical cancer through the AKT signaling pathway, supporting the possibility that SCP3 may be a promising novel cancer target for cervical cancer therapy.

## Introduction

Tumor cells display a variety of antigens including anomalous expression of cancer/testis-associated antigens (CTAs). CTAs are restricted in normal tissues to germ cells of the testis, with occasional expression in female reproductive organs, and are expressed in histologically different types of malignant human tumors [1]–[4]. The tumor restricted expression pattern of CTAs makes them an interesting target for immunotherapeutic approaches [3], [5]. The role of CTA expression in tumor cells remains unclear, and thus is an area of active research. Likewise, our results on the expression of CTAs and their molecular mechanism in tumor cells may provide better insight into tumorigenesis.

Cor1 family members such as X-linked lymphocyte regulated protein (XLR), Xlr-related meiosis regulated protein (XMR), and synaptonemal complex protein 3 (SCP3) are typical CTAs [1]. They are involved in DNA-binding proteins and a structural component of the synaptonemal complex, which mediates synapsis, the pairing of homologous chromosomes during meiosis of germ cells [6]. Notably, male mice deficient for SCP3 are sterile as a result of massive apoptotic cell death in the testis during meiotic prophase [6], [7]. Although SCP3 is expressed strictly in the testis and ovary in normal tissues, expression of SCP3 is frequently observed in various human cancer cells such as acute lymphoblastic leukemia and non-small cell lung cancer [8], [9]. We previously performed a small pilot study, and found that SCP3 is expressed in various cervical cancer cell lines and a small number of cervical cancer tissues [10]. However, none of these studies have provided convincing evidence of the oncogenic potential of SCP3.

To elucidate the role of SCP3 in tumorigenesis, we examined the role of these members as potential oncoproteins by conducting a series of *in vitro* and *in vivo* experiments using both a murine cell line (NIH3T3) and human cervical cancer cell lines (CUMC6, SiHa, CaSki, and HeLa). Here, we report that overexpression of SCP3 induces phenotypic changes characteristic of transformation both *in vitro* and *in vivo*. Furthermore, we analyzed patterns of SCP3 and pAKT expression by immunohistochemistry in cervical tissue specimens from patients with cervical intraepithelial neoplasia (CIN) or invasive cervical carcinoma. The relationships between protein expression and clinicopathological parameters/survival of cervical cancer patients were also analyzed, and demonstrated that SCP3 expression is a prognostic factor for patients with cervical cancer.

## Materials and Methods

### Mice and cell lines

Six- to eight-week-old female Balb/c Nude mice were purchased from Daehan Biolink (Chungbuk, Korea). All animal procedures were performed under a protocol approved by the Korea University Institutional Animal Care and Use Committee (KUIACUC-2009-126). The murine fibroblast cell line NIH3T3 and human cervical cancer cell lines CUMC6, SiHa, CaSki, and HeLa were purchased from American Type Culture Collection (ATCC, Manassas, VA) and were grown in Dulbecco's Modified Eagle Medium (DMEM) in the presence of 10% fetal bovine serum (FBS). All cells were cultured in 5% CO_2_ balanced air at 37°C. The identities of cell lines were confirmed by short tandem repeat (STR) profiling by IDEXX Laboratories Inc. and used within 6 months for testing.

### Patients and tumor samples

Primary tumor specimens were obtained between 1996 and 2010 from 181 cases of cervical cancer, 301 cases of high grade cervical intraepithelial neoplasia (CIN), and 99 cases of low grade CIN undergoing primary surgery at Gangnam Severance Hospital, Yonsei University College of Medicine. All patients gave oral and written informed consent. Paraffin blocks for some of the patients were provided by the Korea Gynecologic Cancer Bank through the Bio & Medical Technology Development Program of the Ministry of Education, Science, and Technology, Korea (http://www.kgcb.or.kr). All tumor tissues were histologically reviewed and only specimens with a sufficient abundance of tumor cells were included in tissue microarray construction. Staging was performed according to the International Federation of Gynecology and Obstetrics (FIGO) staging system. Primary treatment for invasive cervical cancer consisted of a type 3 radical hysterectomy with pelvic lymph node dissection. Concurrent Platinum-based chemoradiation was given in cases with increased risk of recurrence, such as positive resection margins, positive lymph nodes, or parametrial invasion. Medical records were reviewed to obtain data including age, surgical procedure, survival time, and survival status. Response to therapy was assessed according to the Response Evaluation Criteria in Solid Tumors (RECIST; version 1.0), either by computed tomography or magnetic resonance imaging [11]. Data on tumor size, cell type, tumor grade, and lymph node metastasis were obtained by reviewing pathology reports. The study protocol was approved by the Institutional Review Boards (IRBs) of Gangnam Severance Hospital and the Office of Human Subjects Research at the National Institutes of Health (NIH).

### Construct of SCP3 and its deletion mutant expression vectors

hSCP3 full and deletion mutants were created with a PCR-based strategy from a human testis cDNA library (Clontech, Moutain View, CA) with the following primers: hSCP3 1 forward 5′-GCTCGAGATGGTGTCCTCCGGAAAAAAG-3′; hSCP3 81 forward 5′-CCCTCGAGACCATGATTAACAAGGCTCTTCTT-3′; hSCP3 131 forward 5′-GGCTCGAGATGGATATGCAGAAAGCTGAG-3′; hSCP3 80 reverse 5′-CGAATTCTCAGTCAACTCCAACTCCTTCCA-3′; hSCP3 130 reverse 5′- CGAATTCTCAGAAACTGCTGAGAATAT-3′; hSCP3 236 reverse 5′-CGAATTCAGTCTTATTGTACCTAACTTCTCTG-3′. hSCP3 1-236 (full), 1-80, 1-130, 81-236, and 131-236 fragments were subcloned into the pMSCV-puro vector (Clontech) at the *Xho* I and *EcoR* I restriction sites. Recombinant pMSCVs encoding SCP3 or its deletion mutants were confirmed by DNA sequence analysis.

### Construct of shSCP3 vectors

To generate the Scp3 short hairpin RNA (shRNA) exoression construct human Scp3-shRNA annealed forward 5′ GATCCGGAGAAGAATCATGATAATTCAAGAGAT TATCATGATTCTTCTCCTTTTTTGGAAA-3′ (*Bam*HI-compatible) and reverse 5′- AGCTTTTCCAAAAAAGGAGAAGAATCATGATAATCTCTTGAATTATCATGATTCTTCTCCG-3′ (*Hind*III-compatible) oligonucleotides were ligated to *Bam*HI/*Hind*III-digested pSilencer 3.1-H1 puro. The shRNA control employed for these studies was a scrambled DNA sequence that does not target any identified human coding sequence (Ambion, Austin, TX).

### Western blot analysis

For each experiment, a total of 5×10^5^ cells were rinsed twice with ice-cold PBS and added 0.2 mL of the Protein Extraction Solution RIPA (Elpis Biotech, Daejeon, Korea) [50 mM Tris Cl, pH 8.0, 150 mM NaCl, 1 mM phenylmethylsulphonyl fluoride (PMSF), 0.1% sodium dodecyl sulphate (SDS), 1% Nonidet P-40 (NP-40), 0.5 mM EDTA], incubated for 30 min on ice, and then were scraped and centrifuged. Protein concentrations were determined by the coomassie plus protein assay (Pierce, Rockford, USA). 50 µg of protein were solubilized in Laemmli buffer (62.5 mM Tris/HCL pH 6.8, 10% glycerol, 2% SDS, 5% mercaptoethanol and 0.00625% bromophenol blue), boiled for 5 min, and then separated by SDS polyacrylamide gel electrophoresis. Separated gel was transferred to nitrocellulose membranes at 90 V for 1 hr on ice. Primary antibodies against phospho AKT (Ser473), AKT, and phospho ERK (T202/Y204) were purchased from Cell Signaling (Beverly, MA) and used at a dilution of 1∶1000. Likewise, primary antibodies against dual phospho p38 MAPK (Stressgen, Victoria, Canada) and SCP3 (BD Biosciences, San Jose, CA) were used at a dilution of 1∶1000, while antibodies against β-actin and Flag (Sigma-Aldrich, St. Louis, MO) were used at a dilution of 1∶10,000 in Tris-buffered saline (TBS)-T containing 5% BSA (Santa Cruz Biotechnology, Santa Cruz, CA) at 4°C overnight, followed by 3 washes in TBST, 5 minutes per wash. Goat anti-mouse IgG-HRP, anti-rabbit IgG-HRP secondary antibodies (1∶5000) (Stressgen) were incubated for 1 hr at room temperature conjugated with horseradish peroxidase. Immunoreactive bands were visualized by enhanced chemiluminescence (ECL, Elpis Biotech). Densitometry was performed using an image analyzer Fujifilm LAS-4000 (Fuji, Tokyo, Japan) and Multi Gauge V3.1 imaging software (Fujifilm Medical systems USA, Inc., Edison, NJ). To quantitate the intensity profile of protein band images, we used Image J densitometry software (Version 1.6, National Institutes of Health, Bethesda, MD). The band images were scanned in grayscale at a resolution of at least 600dpi and expressed a percentage of the total size of all the measured peaks. To calculate a relative value, the intensity of each sample was divided by that of control. Numbers below western blots refer to the relative values of the intensity normalized to each control.

### Cell proliferation and colony formation assays

For cell proliferation assays, the cells (n = 3) were grown in six well plates at a density of 1×10^4^ cells per well for 3 days. The percentages of live cells were determined using a hematocytometer after 0.4% trypan blue staining. Measurements were made in triplicate for each of the cell lines and the experiments were repeated three times. For the soft-agar colony formation assay, 1×10^4^ NIH3T3 cells, CaSki cells and HeLa cells were plated in 6-well culture plate as a suspension in 2 mL of DMEM containing 10% FBS and 0.4% agar on top of the base layer of 0.7% agar containing 2 mL of the same medium. Plates were incubated at 37°C for 2 weeks until colonies were formed. Two weeks later, >2 mm colonies were counted with an ocular micrometer on a microscope. All experiments repeated at least twice.

### siRNA treatment

Synthetic small interfering RNA (siRNA) specific for green fluorescent protein (GFP) and mouse AKT were purchased from Invitrogen (Carlsbad, CA). The following sequences were used: GFP (non-specific target control) 5′-GCAUCAAGGUGAACUUCAA-3′ (sense), 5′-UUGAAGUUCACCUUGAUGC-3′ (antisense); AKT, 5′-GACAACCGCCAUCCAGACU-3′ (sense), 5′-AGUCUGGAUGGCGGUUGUC-3′ (antisense). For *in vitro* delivery, NIH3T3 cells on a 6-well vessel were transfected with 300 pmol of the synthesized siRNAs using Lipofectamine 2000 (Invitrogen) according to the manufacturer's instructions. The siRNA-treated cells were collected 48 h after transfection for western blot analysis, cell proliferation assay, and colony forming assay.

### Tumor formation

The transfected NIH3T3 cells (1×10^5^ cells/mouse), CaSki cells (1×10^6^ cells/mouse), or HeLa cells (1×10^6^ cells/mouse) were injected subcutaneously into Balb/c Nude mice. Tumor size was measured twice a week for 24 days after tumor cell injection. Each individual tumor size was measured with a caliper, and the tumor volume was calculated using the following formula: tumor volume (mm^3^) = [width×length^2^]/2 [12].

### Immunofluorescent labeling of tumor tissue sections

Tumor tissue samples were fixed and embedded in Tissue-Tek O.C.T. compound (Sakura Finetechnical, Tokyo, Japan) in molds. For immunofluorescent analyses, 10-µm cryosections were placed on poly-L-lysine–coated slides, fixed in 4% paraformaldehyde, and blocked in PBS with 10% normal goat serum (NGS) (NGS/PBS). Tissue sections were incubated with pAKT antibody (1∶100; Cell signaling) for overnight at 4°C. After washing, sections were incubated with the secondary Alexa555-conjugated goat anti–rabbit IgG (1∶1000 in NGS/PBS; Molecular Probes, Eugene, OR) and then mounted in Fluorescent Mounting medium (Dako, Glostrup, Denmark) on glass microscope slides. All images were acquired using a Zeiss LSM 5 Pascal confocal microscope (Carl Zeiss, Jena, Germany). To quantitate the intensity profile of pAKT fluorescence image in tumor tissue sections, we used ImageJ densitometry software. The tumor tissues on slides were scanned in grayscale at a resolution of at least 600dpi. Tumor areas on scanned images were selected more than 10 times from each group with freehand tool and measured the mean grey value. To calculate a relative value, the intensity of each sample was divided by that of blank slides (without primary antibody).

### Tissue microarray construction

Full-face sections of all donor blocks were stained with haematoxylin and eosin (H&E) and reviewed by a pathologist who marked representative tumor areas. The tissue microarray (TMA) was constructed using a Manual Tissue Arrayer MTA-1 (Beecher Instruments Inc., Silver Spring, MD). Four 1.0 mm diameter tissue cores consisting of matched tumor specimens and normal epithelial samples were extracted from selected regions of each donor block. The presence of tumor tissues on the TMA was verified with H&E staining. Multiple 5 µm thick sections were cut with a microtome and H&E staining of TMA slides were examined every 50^th^ section for the presence of tumor cells.

### Immunohistochemistry and scoring

TMA sections were deparaffinized and hydrated in xylene and descending gradient alcohol solutions. Endogenous peroxidase was blocked by incubation in 3% H_2_O_2_ for 10 minutes. Antigen retrieval was performed in a steam pressure cooker (Pascal, Dako, Carpinteria, CA) with prewarmed antigen retrieval buffer pH 9 (Dako) at 95°C for 10 minutes. To minimize nonspecific staining, sections were incubated with protein block (Dako) for 20 minutes. The sections were incubated with an anti-SCP3 antibody (clone 25/SCP3, BD Biosciences, dilution 1∶500) overnight at 4°C or an anti-pAKT antibody (Cell Signaling; dilution 1∶200) for 120 minutes at room temperature. EnVision FLEX+ and EnVision+ Dual Link System (Dako) were used for the detection of SCP3 and pAKT, respectively. Staining was visualized using 3,3′-Diaminobenzidine (DAB) and the sections were counterstained with hematoxylin. Finally, the slides were covered and observed under a light microscope (Axioplot, Carl Zeiss, Jena, Germany). In this study, five conventional whole section slides were carefully selected to validate the immunohistochemical staining performed for SCP3 and pAKT. During this process, staining patterns were thoroughly compared, and some of the patterns matched those of conventional slides from the same case. Furthermore, in designing this study, stromal cells from the whole sections of cercival cancer were selected as internal positive control while the primary antibody was omitted from the negative control.

SCP3 and pAKT staining results were scored based on (a) intensity [categorized as 0 (absent), 1 (weak), 2 (moderate), or 3 (strong)] and (b) the percentage of positive stained epithelial cells [scored as 0 (0% positive), 1 (1–25%), 2 (26–50%), 3 (51–75%), or 4 (>75%)] (Figure S1). An overall protein expression score was calculated by multiplying the intensity and positivity scores (overall score range, 0–12). The IHC score was then dichotomized into high expression (SCP3 IHC score of >7 and pAKT score of >7, respectively), and low expression (IHC score of ≤7 in both SCP3 and pAKT). Receiver operating characteristic (ROC) analysis was used when determining the cut-off values of the SCP3 and pAKT. The sensitivity and specificity for discriminating death or alive was plotted at each IHC score and cut-off value was established to be the point of the ROC curve where the sum of sensitivity and specificity was maximize [13]. Two independent pathologists with experience in tissue microarray analysis examined the slides without any knowledge of the corresponding clinical data.

### Statistical analysis

All data are representative of at least 2 independent experiments. Non-parametric 1-way or 2-way ANOVA was performed with SPSS version 18.0 software (SPSS Inc., Chicago, IL). Comparisons between individual data points were assessed with Student's T-test. Statistical analyses of SCP3 and pAKT expression were performed using the Mann-Whitney test and the Kruskal-Wallis test. The Spearman nonparametric correlation test was used to analyze the relationship between SCP3 and pAKT. Survival curves were estimated by Kaplan-Meier analysis. The log-rank test was used to compare the differences of survival distribution between groups. The multivariate analysis with Cox proportional hazards model was performed to identify independent predictors of disease-free survival in adjustment with relevant clinical covariates such as age, FIGO stage, histologic type, tumor grade, tumor size, and lymph node status. The final model was chosen based on the result of univariable analysis as well as consideration of the clinical or biological importance of the variables. All statistical tests were two-sided, and *P* values<0.05 were considered statistically significant.

## Results

### Ectopic expression of hSCP3 increases proliferation and tumorigenicity of NIH3T3 cells

**Figure 1:**
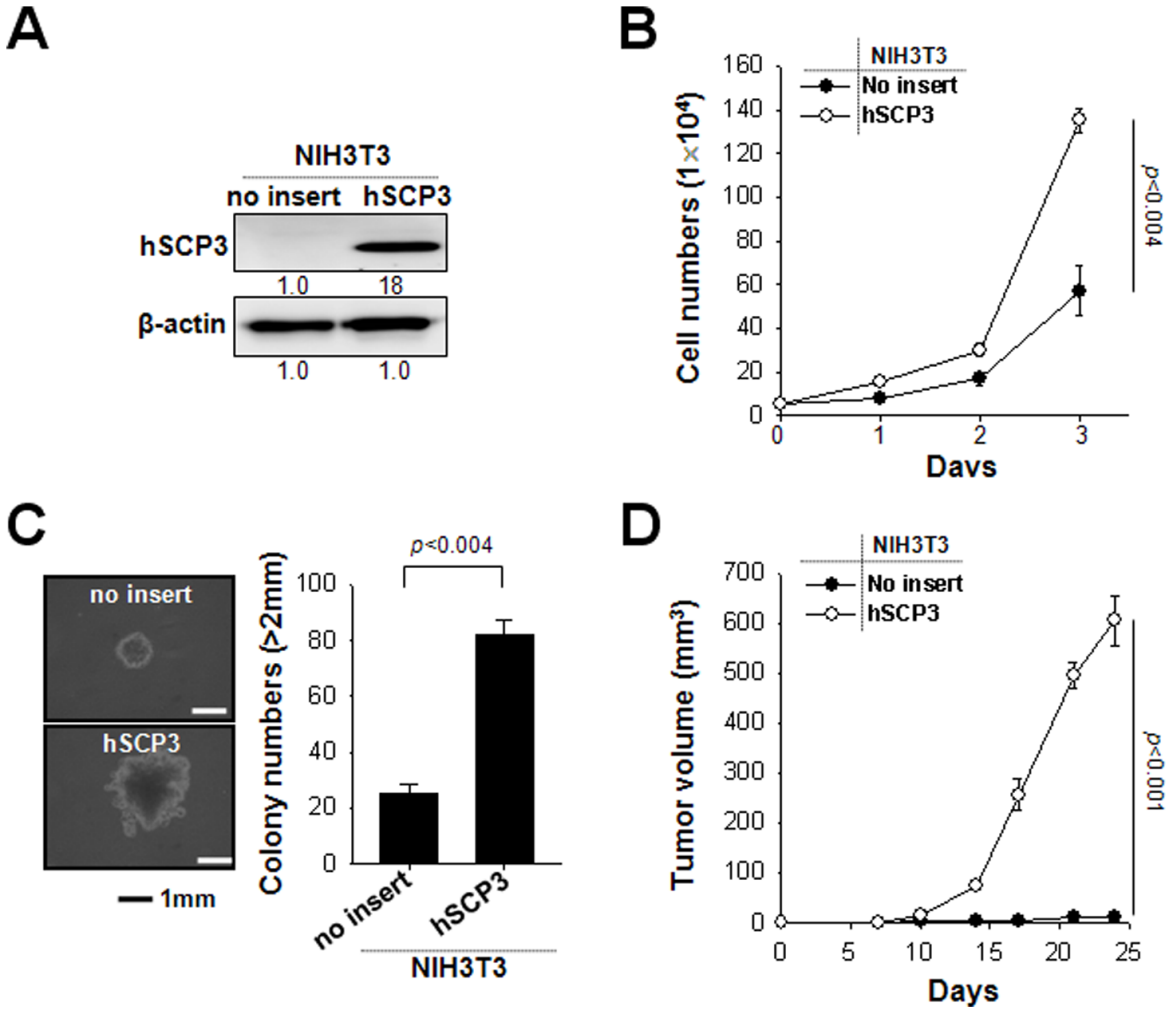
hSCP3 increases oncogenic potentials of NIH3T3 cells *in vitro* and *in vivo*. (A) Western blot analysis of expression of hSCP3 in NIH3T3 cells retrovirally transduced with a pMSCV vector encoding hSCP3 (NIH3T3/hSCP3). NIH3T3/No insert cells were used as a control. Numbers below western blots refer to the relative values of the intensity normalized to no insert control. (B) *In vitro* growth curves of NIH3T3/hSCP3 cells. Cells were counted after trypan blue staining to exclude dead cells. (C) Colony-forming capacity of NIH3T3/hSPC3 cells in soft agar; (left) Representative images of average colony size in each group; (right) Bar graph representing the number of colonies with diameters greater than 2 mm in soft agar (scale bar: 1 mm). (D) Tumorigenicity of NIH3T3/hSCP3 cells. Balb/c Nude mice (n = 5) were inoculated subcutaneously with 1×10^5^ cells/mouse of NIH3T3/no insert or NIH3T3/hSCP3 cells. Error bars represent the mean ± SD.

A previous study reported that human SCP3 (hSCP3) is expressed in various cancer cells and that its expression in human specimens is associated with oncogenesis [8], [10]. However, the oncogenic potential of SCP3 has not been sufficiently characterized at either the molecular or cellular level. To examine whether hSCP3 may have cellular tumorigenic potential, we first used a non-tumorigenic murine fibroblast NIH3T3 cell line and established the cell lines expressing hSCP3 using a retroviral transduction system (NIH3T3/hSCP3). NIH3T3/no insert (empty vector) cells were also established as a control. The expression of SCP3 protein in NIH3T3/hSCP3 cells was confirmed by Western blot (Figure 1A). Cell proliferation of transduced NIH3T3 cells was measured by counting the number of live cells after trypan blue staining. In cell proliferation assays (Figure 1B), NIH3T3/hSCP3 cells exhibited a significantly increased proliferation rate compared with NIH3T3/no insert cells (*P*<0.005). The results of a soft agar colony formation assay with the same cell lines were consistent with those of the cell proliferation assay (Figure 1C). NIH3T3/hSCP3 cells exhibited significantly higher potentials of colony formation in soft agar as compared with NIH3T3/no insert cells (*P*<0.005). We next subcutaneously injected hSCP3- or no insert-expressing NIH3T3 cell in the left flank of athymic Balb/c Nude mice (Figure 1D). Consistent with our *in vitro* data, the tumor-forming ability of NIH3T3 cells was drastically increased by ectopically-expressing hSCP3 (no insert *vs*. hSCP3, *P*<0.001). We next explored whether other genes from the murine Cor1 family such mXLR, mXMR and mSCP3, which share more than 60% homology with hSCP3, could have similar tumorigenic potential in the NIH3T3 model [10]. As shown in Figure S2, ectopic expression of Cor1 family members substantially promoted *in vitro* cell proliferation and soft agar colony formation as well as *in vivo* tumor formation in NIH3T3 cell line model, which was consistent with that of the hSCP3. Taken together, these data clearly demonstrate the oncogenic potentials of SCP3 and other Cor1 members in NIH3T3 model.

### hSCP3 promotes tumorigenesis through AKT pathway in NIH3T3 cells

**Figure 2:**
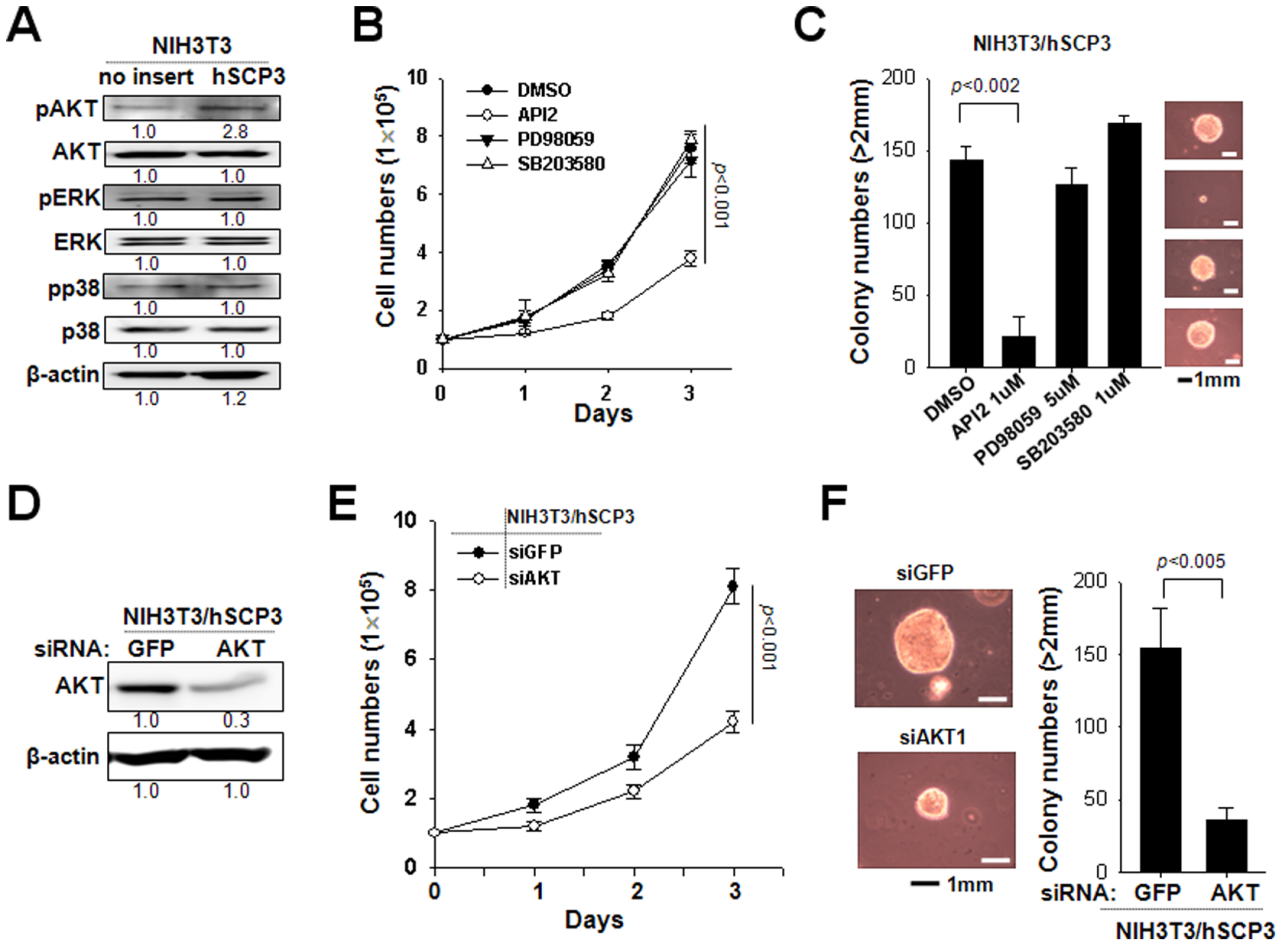
hSCP3 increases tumorigenesis through AKT pathway. (A) Western blot analysis of levels of pAKT, pERK, and pp38 in NIH3T3 cells ectopically expressing hSCP3 or no insert. Numbers below western blots refer to the relative values of the intensity normalized to no insert control. (B) *In vitro* growth curves of NIH3T3/hSCP3 cells treated with DMSO or API2 (Akt inhibitor), PD98059 (Erk inhibitor), or SB203580 (p38 inhibitor). (C) Soft agar colony-forming capacity of NIH3T3/hSPC3 cells in the presence of API2, PD98059, or SB203580; (C, Right) Representative images of average colony size in each group; (C, Left) bar graph showing the number of colonies with diameters greater than 2 mm in soft agar (scale bar: 1 mm). (D) Western blot analysis of levels of pAKT in NIH3T3/hSCP3 cells transfected with a siRNA targeting GFP or AKT (siGFP or siAKT) to confirm the reduction of AKT protein level. Numbers below western blots refer to the relative values of the intensity normalized to no insert control. (E) *In vitro* growth curves of NIH3T3/hSCP3 cells transfected with siGFP or siAKT. (F) Soft agar colony-forming capacity of NIH3T3/hSPC3 cells treated with siGFP or siAKT; (Left) Representative images of average colony size in each group; (Right) Bar graph representing the number of colonies with diameters greater than 2 mm in soft agar (scale bar: 1 mm). Error bars represent the mean ± SD.

We previously reported that pAKT level is increased in hSCP3-expressing cervical cell lines [10]. To evaluate changes in various signaling molecules that may play a role in the global control of cell proliferation, we performed Western blot analysis to measure the levels of Ser 473 phosphorylated AKT, Thr 202/Tyr 204 phosphorylated ERK, and Thr 180/Tyr 182 phosphorylated p38 MAP kinase in NIH3T3 cells expressing hSCP3 or no insert (empty vector). We observed that the expression of pAKT was significantly increased in cells transduced with hSCP3 compared to control cells (Figure 2A). Next, to explore the relationship between hSCP3-mediated tumorigenic potential and the AKT signaling, we compared both cell proliferation and soft agar colony forming capability of NIH3T3/hSCP3 cells in the presence of control (DMSO), 10 µM API2 (an inhibitor of AKT), 50 µM PD98059 (an inhibitor of MEK/ERK) or 10 µM SB203580 (an inhibitor of p38-MAPK) (Figure 2B and 2C). Treatment with PD 98059 and SB203580 did not significantly affect the proliferation and the colony formation capacity of NIH3T3/hSCP3 cells compared with that of DMSO. On the contrary, API2 treated NIH3T3/hSCP3 cells exhibited a significantly decreased proliferation rate compared with DMSO-treated control (*P*<0.001). Consistently, API2-treated NIH3T3/hSCP3 cells also formed smaller colonies, the number of which was almost 5-times less than colonies formed by DMSO-treated NIH3T3/hSCP3 cells. Thus, it is obvious that both proliferation and colony formation in these cells are dependent on the AKT signaling. To exclude potential off-target effects of API2, we performed *in vitro* cell proliferation and soft agar colony formation experiments using siRNA-targeting AKT (siAKT) to block the AKT pathway. siRNA targeting to irrelevant GFP (GFP siRNA) was used as a negative control. Consistent with API2 data, siAKT-treated NIH3T3/hSCP3 cells exhibited a decreased rate of cell proliferation (Figure 2D and 2E) and colony number compared with control siGFP-treated NIH3T3/hSCP3 cells (Figure 2F). Similar AKT dependency was also observed in NIH3T3 cells expressing mXLR, which displayed the most potent tumorigenic potential among members of the murine Cor1 family (Figure S3). Thus, our data demonstrate that SCP3 and the other Cor1 members could confer tumorigenic potentials upon NIH3T3 cell through AKT pathway.

### The C-terminal region of hSCP3 is sufficient to increase pAKT levels and enhance tumorigenesis

**Figure 3:**
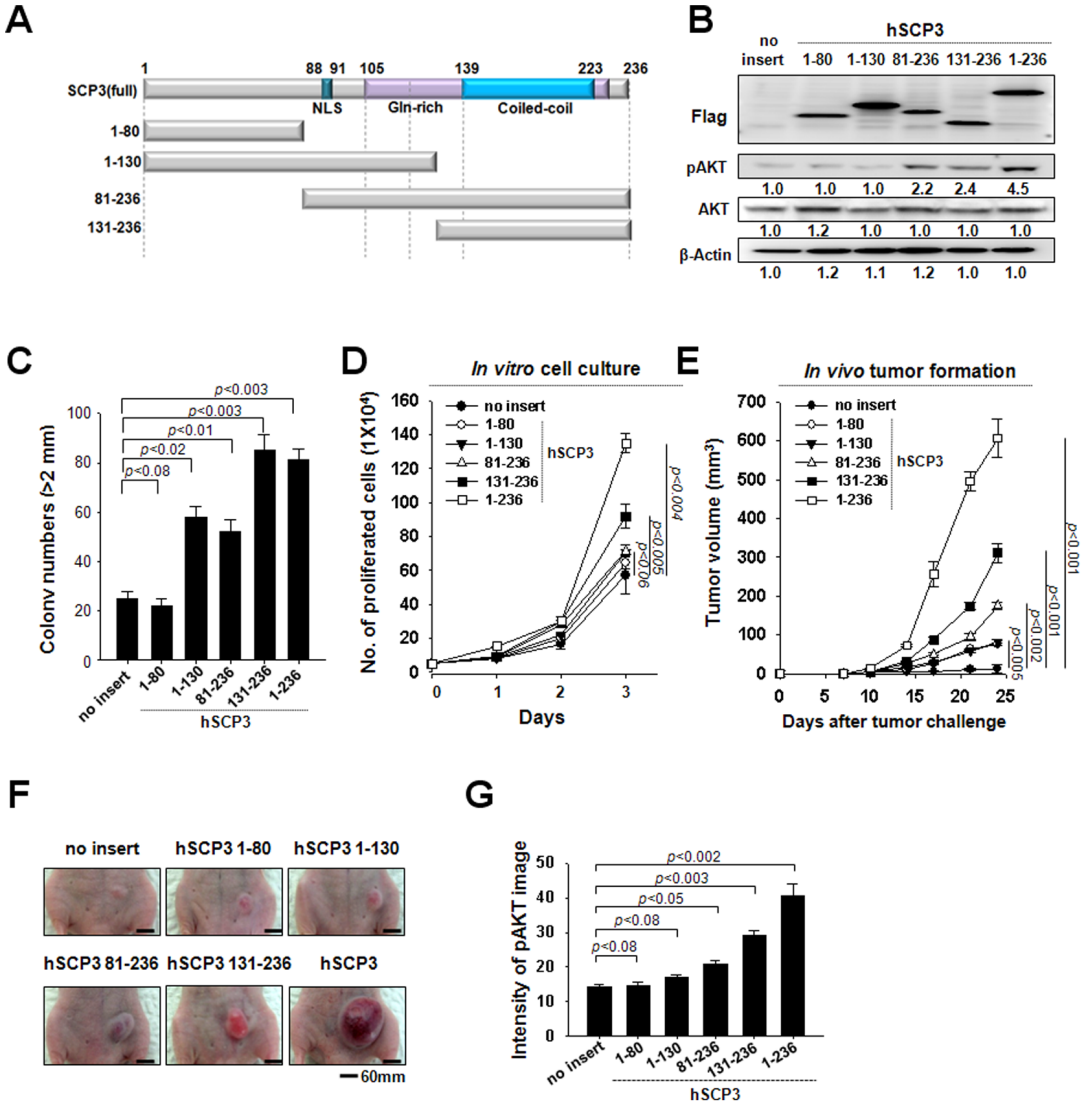
The C-terminal region of hSCP3 is important for its oncogenic capability. (A) Schematic of the domains in hSCP3. Boxes represent the NLS, Gln-rich, and coiled-coil motifs, respectively. (B) Western blot analysis of levels of hSCP3 and its deletion mutants, the N-termini of which were tagged with a Flag epitope for visualization. Numbers below western blots refer to the relative values of the intensity normalized to no insert control. (C) Soft agar colony-forming capacity of NIH3T3 expressing wild type or deletion mutants of hSCP3. (D) *In vitro* growth curves of NIH3T3 cells expressing wild type or deletion mutants of hSCP3. Cells were counted after trypan blue staining to exclude dead cells. (E) *In vivo* tumorigenicity of the NIH3T3 cells. Balb/c Nude mice (n = 5) were inoculated subcutaneously with 1×10^5^ cells/mouse of the NIH3T3 cells expressing full length or mutant hSPC3. Error bars represent the mean ± SD. (F) Representative images of skin tumors (scale bar: 60 mm). (G) Bar graph representing intensity of pAKT fluorescence image on tumor tissue sections, ImageJ densitometry software was used to quantitate the intensity of image as described in Materials and Methods. Error bars represent the mean ± SD.

As schematized in Figure 3A, hSCP3 contains a nuclear localization signal (NLS, residues 88–91), Gln-rich (residues 106–139) and coiled-coil (residues 131–236) motifs. To identify key motifs involved in hSCP3-mediated oncogenesis, we constructed four deletion mutants of hSCP3 containing different motifs (Figure 3A). The N-termini of full hSCP3 and the deletion mutants were tagged with a Flag epitope for visualization by Western blot. We then characterized the expression and the function of hSCP3 and its mutants in retrovirus-transduced NIH3T3 cells. Notably, mutants lacking the coiled-coil motif (hSCP3_1-80_ and hSCP3_1-130_) failed to increase phosphorylation of AKT (Figure 3B). Conversely, hSCP3_81-236_ and hSCP3_131-236_ mutants containing the coiled-coil motif successfully increased levels of pAKT (Figure 3B). Consistent with these observations, NIH3T cells expressing hSCP3_81-236_ or hSCP3_131-236_ exhibited an increased rate of cell proliferation and number of colony compared with those expressing hSCP3_1-80_ and hSCP3_1-130_ as well as no insert (Figure 3C and 3D). More importantly, as shown in Figure 3E-3G, similar phenomena were also observed *in vivo* with tumor xenograft experiments. The tumor-forming ability of NIH3T3 cells was drastically increased after ectopic expression of hSCP3_81-236_ and hSCP3_131-236_ [no insert *vs*. hSCP3_81-236_ (*P*<0.01) or hSCP3_131-236_ (*P*<0.003)]. Similarly, immunofluorescence staining for pAKT was increased substantially in histological sections of tumors ectopically expressing hSCP3_81-236_ or hSCP3_131-236_ [no insert *vs*. hSCP3_81-236_ (*P*<0.05) or hSCP3_131-236_ (*P*<0.003)] (Figure 3G). Taken together, our data indicate that the coiled-coil motif containing the C-terminal region is the primary contributor to the AKT-dependent oncogenic potential of hSCP3.

### SCP3 has a key role in proliferation and tumorigenicity of cervical cancer cells

**Figure 4:**
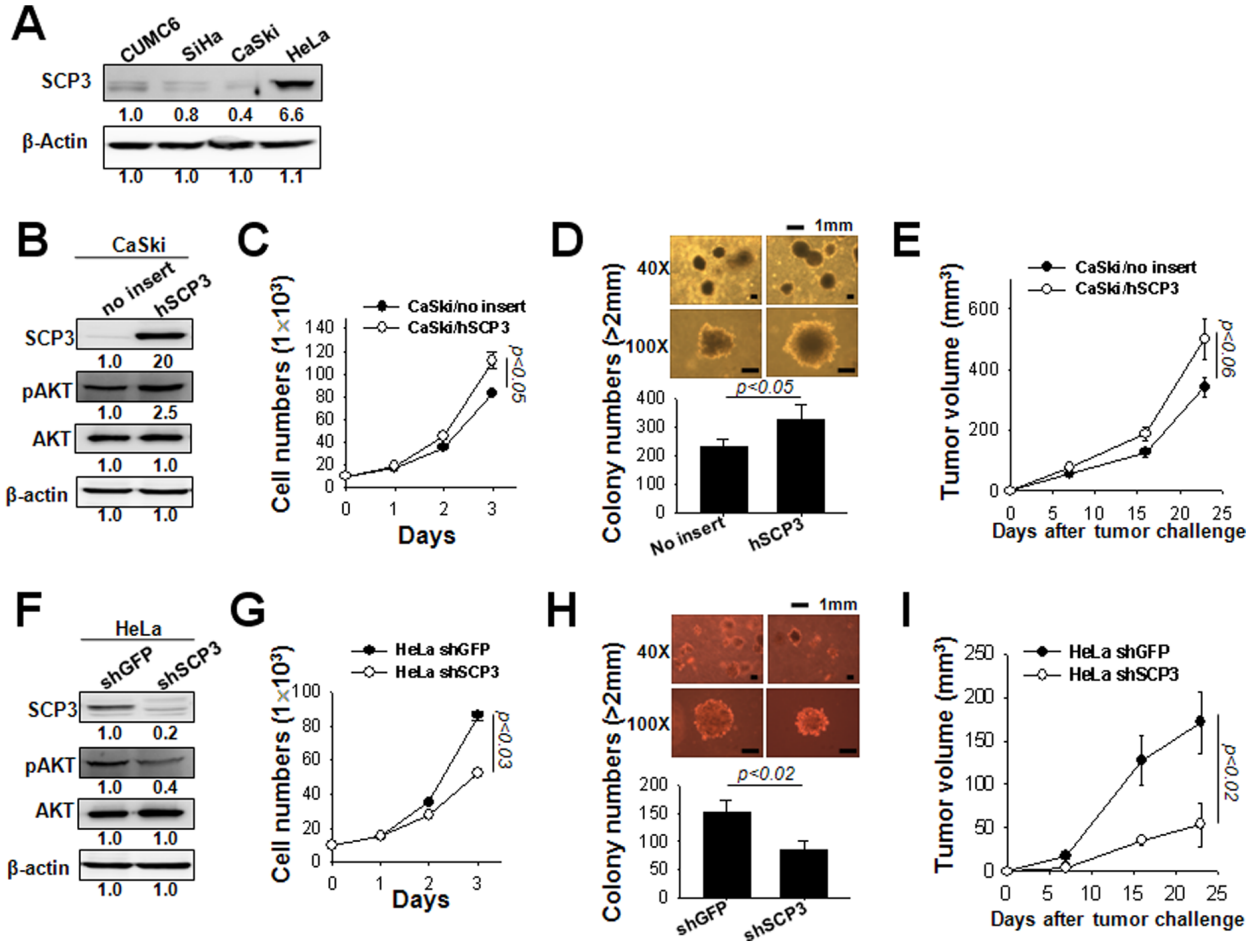
hSCP3 increases oncogenic potentials of human cervical cancer cells *in vitro* and *in vivo*. (A) Western blot analysis of levels of SCP3 in human cervical cancer cell lines CUMC6, SiHa, CaSki, and HeLa cells. Numbers below western blots refer to the relative values of the intensity normalized to CUMC6 control. (B) Western blot analysis of expression of SCP3, pAKT, and AKT in CaSki cells retrovirally transduced with a pMSCV vector encoding hSCP3 (CaSki/hSCP3). CaSki/no insert cells were used as a control. Numbers below western blots refer to the relative values of the intensity normalized to no insert control. (C) *In vitro* growth curves of CaSki/hSCP3 cells. Cells were counted after trypan blue staining to exclude dead cells. (D) Colony-forming capacity of CaSki/hSCP3 cells in soft agar; (Top) Representative colony images of each group; (Bottom) Bar graph representing the number of colonies with diameters greater than 2 mm in soft agar (scale bar: 1 mm). (E) Tumorigenicity of CaSki/hSCP3 cells. Balb/c Nude mice (n = 5) were inoculated with CaSki/no insert or CaSki/hSCP3 cells (1×10^6^ cells/mouse) subcutaneously. (F) Western blot analysis of expression of SCP3, pAKT, AKT in HeLa/shSCP3 cells. HeLa/shGFP were used as a control. Numbers below western blots refer to the relative values of the intensity normalized to shGFP control. (G) *In vitro* growth curves of HeLa/shSCP3 cells. (H) Colony-forming capacity of HeLa/hSCP3 cells in soft agar (scale bar: 1 mm). (I) Tumorigenicity of HeLa/shSCP3 cells. Balb/c Nude mice (n = 5) were inoculated subcutaneously with 1×10^6^ cells/mouse of HeLa/shGFP or HeLa/shSCP3 cells. Error bars represent the mean ± SD.

We further investigated the oncogenic role of SCP3 and its relationship with phosphorylated AKT (pAKT) in human cervical cancer cell lines. For this, the expression of SCP3 was measured in human cervical cancer cell lines CUMC6, SiHa, CaSki, and HeLa using western blot analysis. As shown in Figure 4A, expression of SCP3 was observed in all of cervical cancer cells although that of SCP3 was most profound in HeLa cells. In contrast, CaSki cells exhibited lowest expression of SCP3 protein. Next, to evaluate the effects of SCP3 on cell proliferation and tumorigenicity in cervical cancer cells, we established the CaSki cell lines expressing hSCP3 or no insert (empty vector) using a retroviral transduction system CaSki/hSCP3 or CaSki/no insert, respectively. Conversely, we established the HeLa cell lines expressing shRNA targeting SCP3 (shSCP3) or GFP (irrelevant negative control, shGFP) using a shRNA expressing vector (pSilencer 3.1-H1 puro), HeLa/shSCP3 or HeLa/shGFP, respectively, after puromycin selection. First of all, SCP3, pAKT, AKT protein levels in the CaSki cells were measured by western blotting. As shown in Figure 4B, the expression of pAKT was significantly increased in CaSki/SCP3 cells compared with control CaSki/no insert cells. The cell growth assay revealed that cell growth rate in the SCP3-transduced cells was significantly higher than control cells (Figure 4C). Similar increase was also observed in colony formation assay (Figure 4D). Consistent with *in vitro* data, the tumor-forming ability of CaSki cells was significantly increased after introducing SCP3 (Figure 4E). In contrast, knock-down of SCP3 in HeLa cells significantly decreased pAKT level, *in vitro* cell growth rate and colony formation efficacies and *in vivo* tumor growth rate compared with shGFP control group (Figure 4F–4I). These data demonstrate that SCP3 has a key role in cell proliferation and tumorigenicity of cervical cancer cells.

### SCP3 and pAKT are overexpressed in tumor samples from patients with cervical cancer

Having elucidated the molecular mechanism by which SCP3 activation promotes tumor growth via the AKT pathway, we next examined SCP3 and pAKT protein expression levels by immunohistochemistry in cervical tissue specimens from patients with CIN or invasive cervical cancer. Table S1 summarizes patient's clinicopathological characteristics. In 181 patients with cervical cancer, 118 patients of stage I, 54 of stage II and 9 of stage IV were included. The ages of the patients ranged from 19 to 83 years (mean, 42.4 years). The tumor sizes ranged from 0.2 to 12 cm (mean, 2.9 cm).

**Figure 5:**
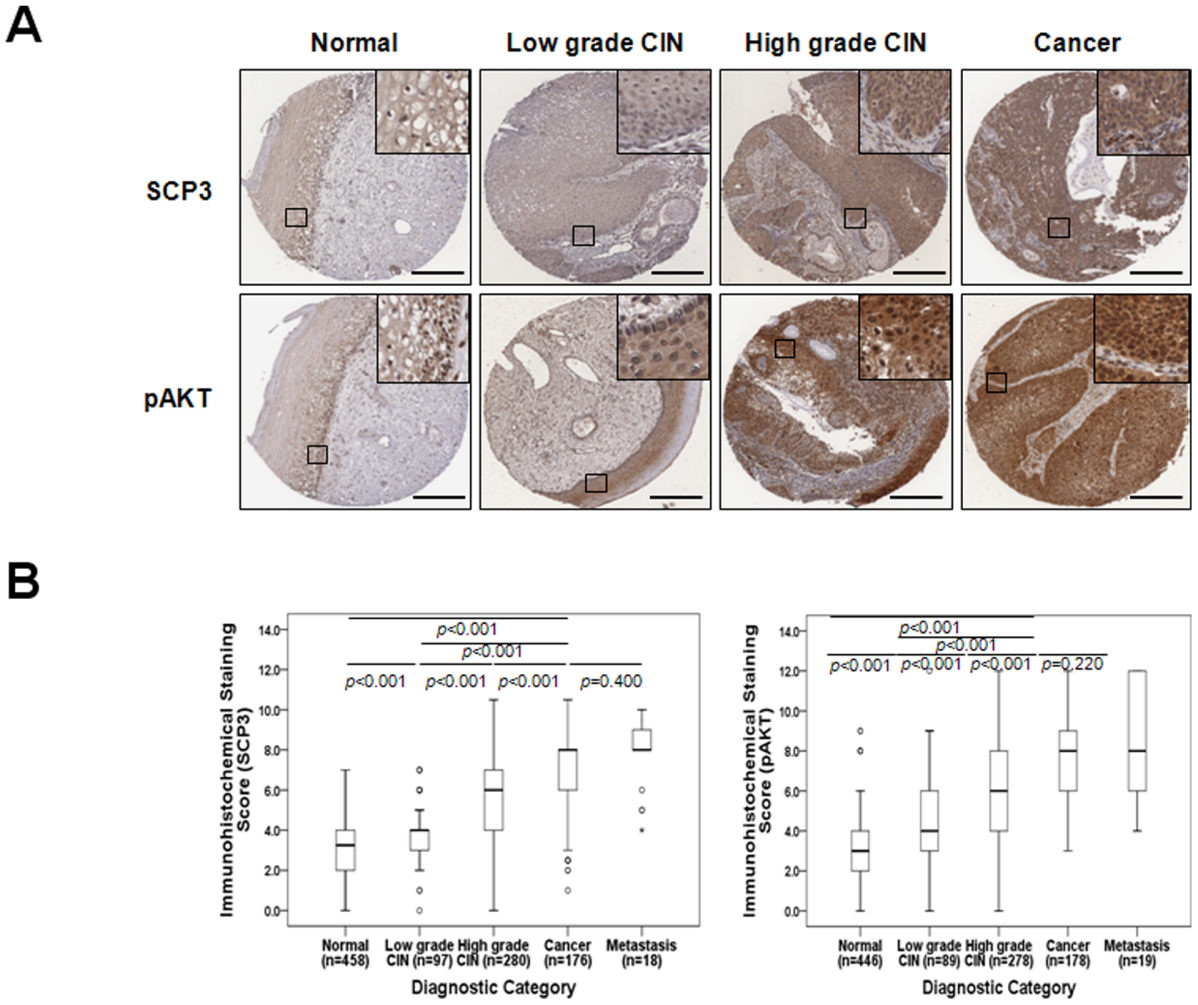
SCP3 overexpression is associated with tumor progression in human cervical neoplasia specimens. (A) Representative immunohistochemical staining images of SCP3 and pAKT in cervical tissue from patients with low-grade CIN, High-grade CIN, and cervical carcinoma. Boxed regions are displayed at high magnification in insets (scale bar: 300 µm). (B) Box plot depiction of IHC staining scores. There was an increasing amount of SCP3 and pAKT expression as tumor stage progressed from low-grade CIN to high-grade CIN to cancer. Symbols indicate individual samples. Numbers associated with symbols indicate case numbers.

Representative immunohistochemical staining of SCP3 and pAKT are shown in Figure 5A. Immunohistochemical staining of SCP3 was observed in the cytoplasm of tumor cells as previously reported in non-small cell lung cancer [8]. The TMA constructed in this study consisted of 181 cases of cervical cancer, however due to the complexity of sectioning, staining, as well as heterogeneity of the samples, 176 (97.2%, SCP3) and 178 (98.3%, pAKT) of which were suitable for IHC evaluation. Detailed IHC scoring patterns are shown in Table S2. A total of 108 of 176 cancers (61.4%) had high expression (cut-off value: 7) of SCP3 whereas 100 of 178 cancers (56.2%) had elevated expression (cut-off value: 7) of pAKT. The level of SCP3 and pAKT expression increased as tumor state progressed from low-grade CIN to high-grade CIN to cancer (Figure 5A and 5B; Table S2). Moreover, SCP3 expression was significantly correlated with FIGO stage (*P* = 0.002), differentiation (*P*<0.001) and chemoradiation response (*P* = 0.005). However, there was no statistically significant difference between pAKT expression and clinicopathologic factors.

We next examined the association between SCP3 and pAKT expression. Of the 400 CIN specimens, 344 (86.0%) were available to confirm co-expression between SCP3 and pAKT, whereas 173 tumor samples (95.6%) were available to confirm co-expression between SCP3 and pAKT in cervical cancer. Notably, expression of SCP3 showed a positive linear correlation with that of pAKT both in CIN (Spearman's rho = 0.322, *P*<0.001) and cervical cancer (Spearman's rho = 0.221, *P* = 0.010) (Table 1, Figure S4).Taken together, these results suggest that SCP3 expression may be involved in the development of cervical cancer development and linked with the AKT signaling pathway.

**Table 1:** Association between SCP3 and pAKT expression in CIN and cervical cancer.

	SCP3 expression						
	Low	%	High	%	Total No.	Correlation coefficient (*r*)	*p* value
**pAKT** **expression**							
*** CIN***						0.322	<0.001
Low	233	90.7	24	9.3	257		
High	62	71.3	25	28.7	87		
*** Cervical cancer***						0.221	0.010
Low	38	50.0	38	50.0	76		
High	29	29.9	68	70.1	97		

CIN, cervical intraepithelial neoplasia.

### SCP3 overexpression is correlated with poor prognosis of patients with cervical cancer

**Figure 6:**
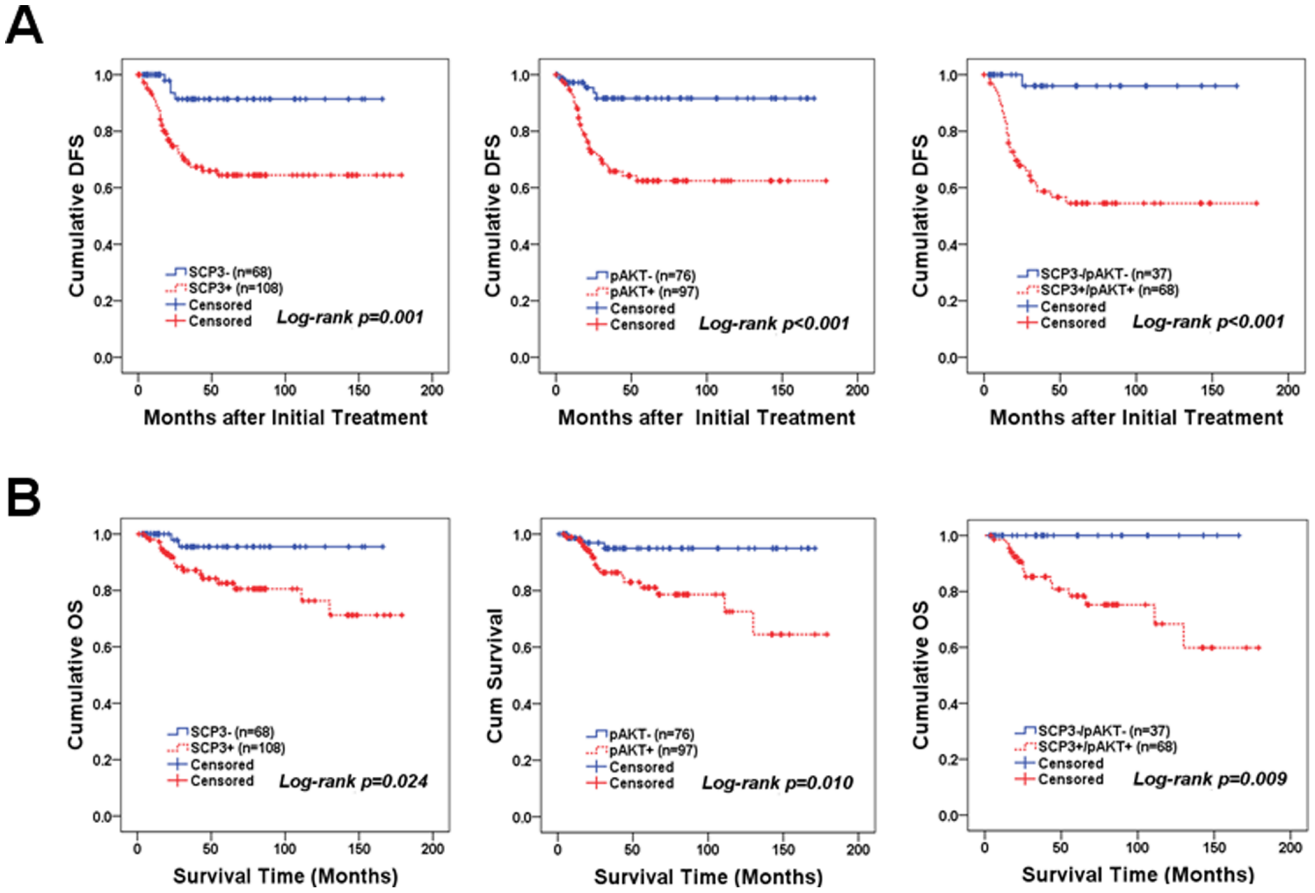
Kaplan-Meier plots for disease-free survival (A) and overall survival (B) according to SCP3 expression, combined SCP3 and pAKT expression, and pAKT expression alone. For patients with high SCP3 expression, the mean disease-free survival and overall survival were 122.4 months and 144.8 months (n = 108), respectively. For patients with low SCP3 expression, mean disease-free survival and overall survival were 153.5 months and 159.6 months (n = 68), respectively.

To investigate the clinical relevance of SCP3, we examined the effect of SCP3 expression on patient outcomes. Five-year disease-free survival and overall survival were analyzed through Kaplan-Meier plots as shown in Figure 6A and B. In survival analysis of SCP3, there were 33 cases of recurrences, 2 cases of persistent disease, and 18 deaths in the 108 patients with high expression of SCP3, whereas low expression of SCP3 (68 patients) was associated with 4 cases of recurrence and 2 deaths over a mean follow-up period of 56.32 months. Patients with high SCP3 expression displayed shorter disease-free survival (mean of 122.4 versus 153.5 months, *P* = 0.001) and overall survival (mean of 144.8 versus 159.6 months, *P* = 0.024) compared with that of patients with low expression of SCP3 (Figure 6A and 6B). Furthermore, patients with combined high SCP3 and high pAKT exhibited significantly worse disease-free survival (mean of 106.7 versus 160.3 months, *P*<0.001) and overall survival (*P* = 0.009) compared with the combined low SCP3 and low pAKT group. Interestingly, there were no deaths in combined low SCP3 and low pAKT patients (n = 37). We next examined the independent prognostic significance of SCP3 alone and high SCP3 combined with high pAKT, as well as other clinicopathological parameters, using the Cox proportional hazards model. According to multivariate analysis, FIGO stage was a significant risk factor for overall survival (*P* = 0.013), whereas lymph node metastasis remained a significant risk factor for disease-free survival (*P* = 0.024) (Table 2). High SCP3 expression was a risk factor for recurrence [hazard ratio = 5.52 (95% CI, 1.78–17.11), *P* = 0.003], whereas high pAKT expression was a risk factor for disease-free survival [hazard ratio = 3.62 (95% CI, 1.19–11.05), *P* = 0.023] and overall survival [hazard ratio = 3.60 (95% CI, 1.04–12.42), *P* = 0.043]. Notably, the combination of high SCP3 and high pAKT expression was a significant risk factor for both disease free survival [hazard ratio = 4.98 (95% CI, 1.90–13.01), *P* = 0.001] and overall survival [hazard ratio = 3.38 (95% CI, 1.21–9.43), *P* = 0.020].

**Table 2:** Univariate and multivariate analyses of the associations between prognostic variables and disease-free survival or overall survival in cervical cancer.

	Disease-free Survival hazard ratio [95% CI], *P* value		Overall Survival hazard ratio [95% CI], *P* value	
	Univariate analysis	Multivariate analysis	Univariate analysis	Multivariate analysis
Age	0.99 [0.97–1.02], 0.923	NA	0.98 [0.94–1.03], 0.583	NA
FIGO stage	5.32 [2.76–10.24], <0.001	1.88 [0.79–4.46], 0.153	3.27 [1.32–8.07], 0.010	3.26 [1.28–8.31], 0.013
Cell type (non-SCC)	1.15 [0.50–2.63], 0.732	NA	2.68 [1.06–6.76], 0.035	2.72 [1.06–6.98], 0.037
Tumor grade (poor)	1.98 [1.03–3.81], 0.040	1.64 [0.76–3.53], 0.207	2.40 [0.96–5.97], 0.060	NA
Tumor size (>4 cm)	2.43 [1.27–4.65], 0.007	1.93 [0.84–4.40], 0.117	1.83 [0.74–4.54], 0.188	NA
LN metastasis	4.97 [2.38–10.38], <0.001	2.57 [1.13–5.87], 0.024	2.23 [0.76–6.47], 0.141	NA
SCC Ag+	1.45 [0.71–2.96], 0.307	NA	1.80 [0.69–4.68], 0.225	NA
SCP3+	5.14 [1.82–14.52], 0.002	5.52 [1.78–17.11], 0.003	4.63 [1.07–20.01], 0.040	3.50 [0.79–15.44], 0.097
pAKT+	4.89 [1.90–12.58], 0.001	3.62 [1.19–11.05], 0.023	4.34 [1.27–14.86], 0.019	3.60 [1.04–12.42], 0.043
SCP3+/pAKT+	4.97 [2.34–10.53], <0.001	4.98 [1.90–13.01], 0.001	4.16 [1.51–11.46], 0.006	3.38 [1.21–9.43], 0.020

CI, confidence interval; FIGO, International Federation of Gynecology and Obstetrics; SCC, squamous cell carcinoma; LN, lymph node; NA, not applicable.

## Discussion

There is increasing evidence that hSPC3 may play a role in oncogenesis; however, the evidence has not been addressed at a cellular or molecular level. In this report, we provide the first compelling data that hSCP3 can play a key role in human tumorigenesis. We demonstrate that ectopic expression of hSCP3 in conventional non-tumorigenic murine NIH3T3 cells and a human cervical cancer cell line CaSki cells leads to increased cell proliferation and colony formation *in vitro* as well as increased tumor growth *in vivo* using a mouse model system. Furthermore, we observed activation of the AKT pathway in hSCP3-overexpressing cells. Interestingly, AKT activation appeared to be associated with hSCP3-mediated oncogenic potential, which was confirmed using an API-2 chemical inhibitor, siAKT treatment, and deletion mutant analysis.

The AKT pathway plays a pivotal role in transformation by inducing cell survival, growth, migration, and angiogenesis [13]–[17]._ENREF_13 Recently, we reported that increased activation of AKT is associated with multiple resistance of tumor cells to various cancer drugs, radiation, as well as cytotoxic T lymphocyte (CTL)-mediated cell death [18]–[21]. In this paper, we demonstrated that hSCP3-mediated oncogenic potentials were primarily mediated by an AKT-dependent pathway. Likewise, it was not surprising that similar AKT dependency was present for murine Cor1 members as demonstrated in Figure S1 and S2, because they share more than 60% homology with hSCP3 [10]. However, we don't exclude the possibility of AKT-independent mechanism in SCP3-mediated oncogenesis since its hSCP3_1-80_ deletion mutant exhibited a significant increase of colony number, proliferation, and tumor volume despite the lack of increased Akt phosphorylation in a NIH 3T3 tumor cell model as shown in Figure 3. Further studies are needed to demonstrate the direct relationship between SCP3 expression and activation of AKT in cervical cancer.

SCP3 is well known as a nuclear protein in meiotic germ cells [22], whereas altered subcellular localization of SCP3 has been reported in various cancer cells as well as human lung cancer tissue specimens [8]. Furthermore, in a previous pilot study using a limited number of cervical cancer specimens, we observed cytoplasmic localization of SCP3 by IHC [10]. The ectopic expression of tumor suppressor proteins can disrupt normal cell differentiation programs and accelerate cancer progression. Therefore, our findings suggest that the cytoplasmic expression of SCP3 may be a cancer-specific phenomenon linked to cancer progression by an unknown mechanism. In the current study, we confirmed that the percentage of patients expressing SCP3 in cervical cancer was relatively high (61.4% of all cases), and was significantly associated with the progression of cervical carcinogenesis from low-grade CIN to high-grade CIN to cancer (Table S1, *P*<0.001). To the best of our knowledge, this is the first report to show that SCP3 protein expression increases according to severity of cervical lesions. Moreover, we observed that SCP3 protein expression was significantly associated with advanced tumor stage (*P* = 0.002), poor tumor grade (*P*<0.001), and poor response to chemoradiation therapy (*P* = 0.005), and exhibited the highest levels of expression in metastatic tissue specimens (Table S1). Notably, consistent with our previous report in non-small cell lung cancer [8], high SCP3 expression in cervical cancer conferred a significantly shorter survival time, suggesting that it may be a potential prognostic predictor for cervical cancer. Based on Cox multivariate analysis, SCP3+, pAKT+, SCP3+/pAKT+, and lymph node metastasis was associated with increased risk of recurrence of cervical cancer. Moreover, SCP3+/pAKT+ was the only independent prognostic factor for both disease-free and overall survival according to multivariate analysis (Table 2). Taken together, these results strongly implicate SCP3 as an important aspect in the pathogenesis of cervical cancer. Likewise, evaluation of SCP3 expression in cervical cancer may be useful for prediction of chemoradiation response, stratified prognosis, and determining follow-up strategies.

Lastly, we examined the correlation of SCP3 expression with pAKT expression in CIN and invasive cervical cancer tissues. Consistent with our *in vitro* data, SCP3 expression was positively associated with pAKT expression in both CIN (*P*<0.001) and cervical cancer (*P* = 0.010) (Table 1). Even when SCP3 expression was compared with levels of pAKT using Spearman nonparametric correlation test, SCP3 expression was significantly associated with pAKT expression in both CIN (Spearman's rho = 0.322, *P*<0.001) and cancer specimens (Spearman's rho = 0.197, *P* = 0.010). Together, these data indicated that SCP3 mediates an oncogenic phenotype of cervical cancer cells through an AKT-dependent pathway. Collectively, the results of this study provide the first molecular and cellular evidence of AKT-dependent oncogenic properties of hSCP3, which may serve as a novel therapeutic target for cervical cancer therapy.

## Supporting Information

Figure S1 Evaluation of SCP3 and pAKT IHC staining. Representative immunohistochemical staining images of the staining intensity (A) weak staining, 1; (B) moderate staining, 2; or (C) strong positive staining in most cells, 3 and the percentage of positive stained epithelial cells (D) 1–25% cells staining positive, 1; (E) 26–50% cells staining positive, 2; (F) 51–75% cells staining positive, 3; or (G) more than 75% cells staining positive, 4. Scale bar: 30 µm (A–C), 100 µm (D–G).
(TIF)

Figure S2 Members of the Cor1 family have oncogenic potential. (A) NIH3T3 cells retrovirally transduced with pMSCV vector encoding either no insert (NIH3T3/no insert), mSCP3 (NIH3T3/mSCP3), mXMR (NIH3T3/mXMR), or mXLR (NIH3T3/mXLR) were analyzed for cellular expression of Cor1 members tagged with Flag by Western blot. (B) *In vitro* growth curves of NIH3T3 cells expressing each of the Cor1 members. NIH3T3/no insert cells were used as a control. The cells were counted after trypan blue staining to exclude dead cells. (C) Bar graph representing the number of colonies with a diameter greater than 2 mm in soft agar. (D) Representative colony images of each group (scale bar: 1 mm). (E) Tumorigenicity of NIH3T3 cells expressing each of the Cor1 members. Balb/c Nude (n = 5) mice were inoculated subcutaneously with 1×10^5^ cells/mouse using NIH3T3/no insert, NIH3T3/mSCP3, NIH3T3/mXMR, or NIH3T3/mXLR cells. Tumor volumes were measured beginning 28 days after tumor inoculation. (F) Representative tumor images of each group.
(TIF)

Figure S3 Oncogenesis by Cor1 family is AKT-dependent. (A) Western blot analysis of levels of pAKT in NIH3T3/no insert, NIH3T3/mSCP3, NIH3T3/mXMR, or NIH3T3/mXLR cells. (B) Soft agar colony-forming capacity of NIH3T3/mXLR cells in the presence of API2 (Akt inhibitor), PD98059 (Erk inhibitor) or SB203580 (p38 inhibitor); (Left) Representative colony images of each group; (Right) Bar graph representing the number of colonies with a diameter greater than 2 mm in soft agar (scale bar: 1 mm). (C) Western blot analysis of levels of pAKT in NIH3T3/hSCP3 cells transfected with siRNA targeting GFP or AKT (siGFP or siAKT) to confirm the reduction of protein levels of AKT. (D) *In vitro* growth curves and (E) *in vitro* soft agar colony formation of siGFP or siAKT-transfected NIH3T3/mXLR cells. Error bars represent the mean ± SD.
(TIF)

Figure S4 Relationship between the SCP3 and pAKT expression in CIN (left panel) and cancer (right panel) samples. The values of pAKT IHC expressions were plotted on the y-axis against the IHC scores of SCP3 on the x-axis.
(TIF)

Table S1 Characteristics of Patients.
(DOCX)

Table S2 Expression of SCP3 and pAKT in relation to clinicopathological characteristics in IHC analysis.
(DOCX)
