# Supplementary figures and images for: Synaptonemal Complex Protein 3 Is a Prognostic Marker in Cervical Cancer
Source: PLoS One. 2014 Jun 6;9(6):e98712. doi: 10.1371/journal.pone.0098712 (PMC4048308; doi:10.1371/journal.pone.0098712)

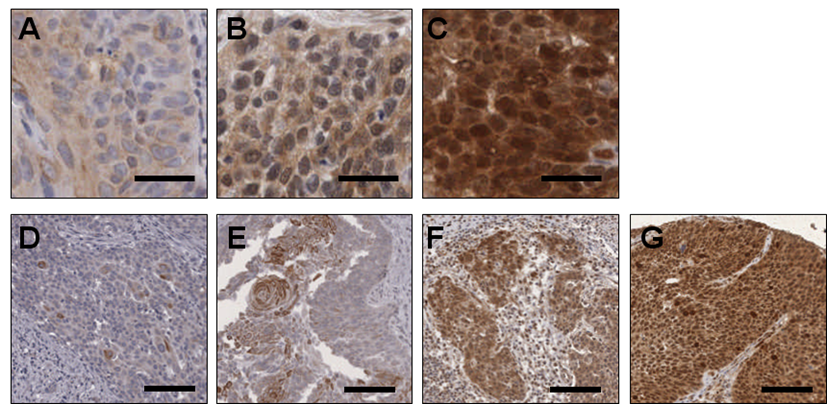

Supplement: Figure S1 — Evaluation of SCP3 and pAKT IHC staining. Representative immunohistochemical staining images of the staining intensity (A) weak staining, 1; (B) moderate staining, 2; or (C) strong positive staining in most cells, 3 and the percentage of positive stained epithelial cells (D) 1–25% cells staining positive, 1; (E) 26–50% cells staining positive, 2; (F) 51–75% cells staining positive, 3; or (G) more than 75% cells staining positive, 4. Scale bar: 30 µm (A–C), 100 µm (D–G). (TIF) [file pone.0098712.s001.tif]

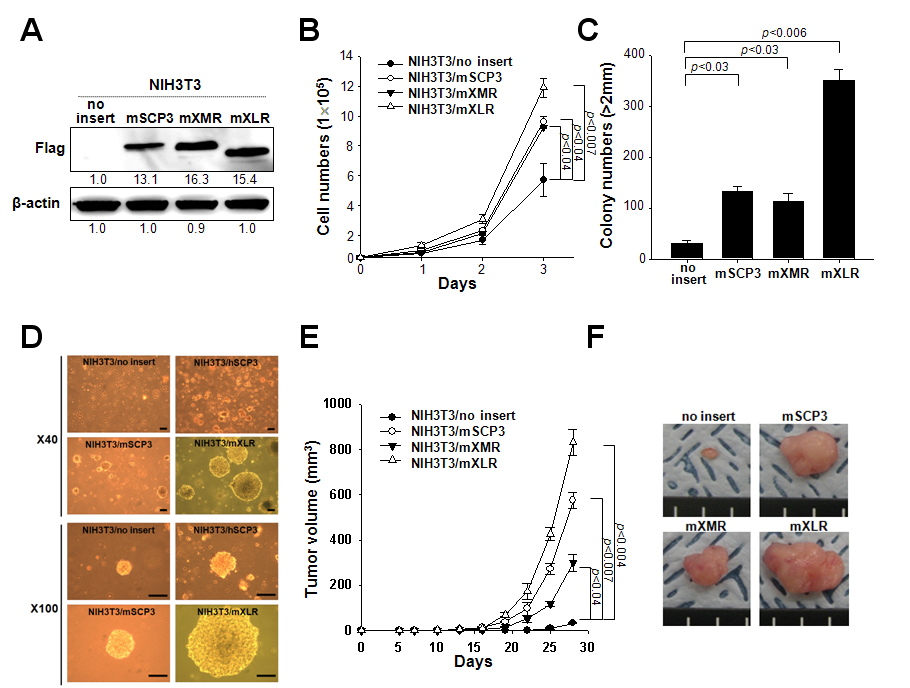

Supplement: Figure S2 — Members of the Cor1 family have oncogenic potential. (A) NIH3T3 cells retrovirally transduced with pMSCV vector encoding either no insert (NIH3T3/no insert), mSCP3 (NIH3T3/mSCP3), mXMR (NIH3T3/mXMR), or mXLR (NIH3T3/mXLR) were analyzed for cellular expression of Cor1 members tagged with Flag by Western blot. (B) In vitro growth curves of NIH3T3 cells expressing each of the Cor1 members. NIH3T3/no insert cells were used as a control. The cells were counted after trypan blue staining to exclude dead cells. (C) Bar graph representing the number of colonies with a diameter greater than 2 mm in soft agar. (D) Representative colony images of each group (scale bar: 1 mm). (E) Tumorigenicity of NIH3T3 cells expressing each of the Cor1 members. Balb/c Nude (n = 5) mice were inoculated subcutaneously with 1×10^5^ cells/mouse using NIH3T3/no insert, NIH3T3/mSCP3, NIH3T3/mXMR, or NIH3T3/mXLR cells. Tumor volumes were measured beginning 28 days after tumor inoculation. (F) Representative tumor images of each group. (TIF) [file pone.0098712.s002.tif]

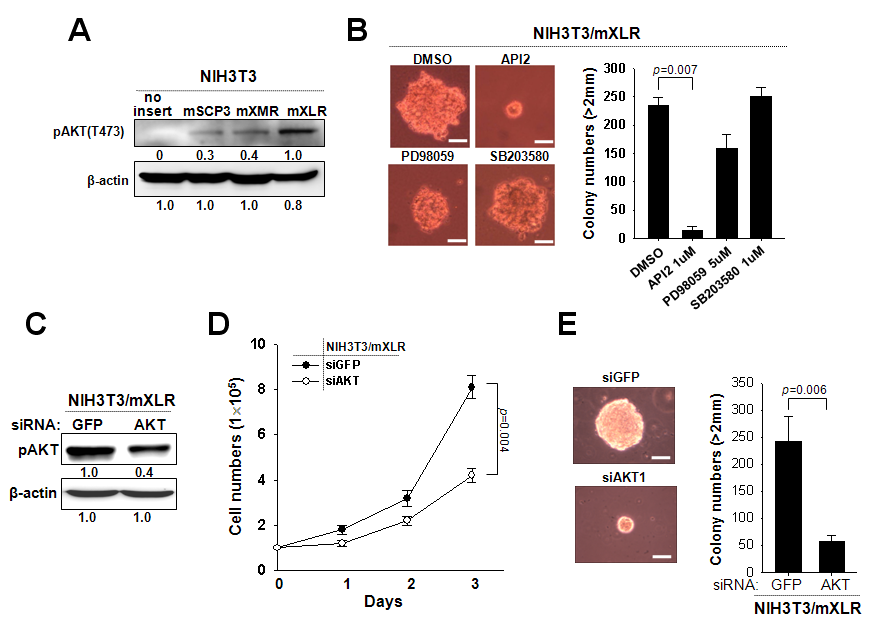

Supplement: Figure S3 — Oncogenesis by Cor1 family is AKT-dependent. (A) Western blot analysis of levels of pAKT in NIH3T3/no insert, NIH3T3/mSCP3, NIH3T3/mXMR, or NIH3T3/mXLR cells. (B) Soft agar colony-forming capacity of NIH3T3/mXLR cells in the presence of API2 (Akt inhibitor), PD98059 (Erk inhibitor) or SB203580 (p38 inhibitor); (Left) Representative colony images of each group; (Right) Bar graph representing the number of colonies with a diameter greater than 2 mm in soft agar (scale bar: 1 mm). (C) Western blot analysis of levels of pAKT in NIH3T3/hSCP3 cells transfected with siRNA targeting GFP or AKT (siGFP or siAKT) to confirm the reduction of protein levels of AKT. (D) In vitro growth curves and (E) in vitro soft agar colony formation of siGFP or siAKT-transfected NIH3T3/mXLR cells. Error bars represent the mean ± SD. (TIF) [file pone.0098712.s003.tif]

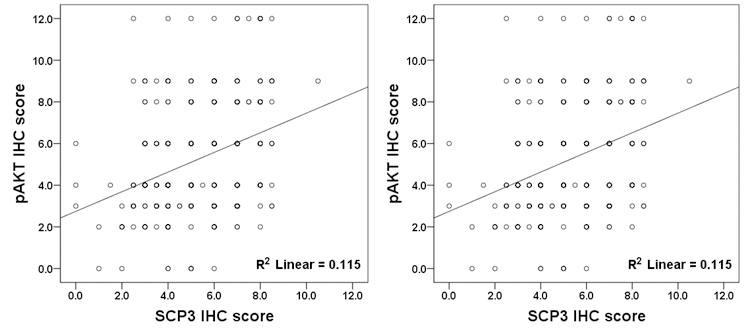

Supplement: Figure S4 — Relationship between the SCP3 and pAKT expression in CIN (left panel) and cancer (right panel) samples. The values of pAKT IHC expressions were plotted on the y-axis against the IHC scores of SCP3 on the x-axis. (TIF) [file pone.0098712.s004.tif]
