# Supplementary material for: Synaptonemal Complex Protein 3 Is a Prognostic Marker in Cervical Cancer
Source: PLoS One. 2014 Jun 6;9(6):e98712. doi: 10.1371/journal.pone.0098712 (PMC4048308; doi:10.1371/journal.pone.0098712)
Supplement: Table S1 — Characteristics of Patients. (DOCX) [file pone.0098712.s005.docx]

**Table S1. Characteristics of Patients**

| **Variable** | **Frequency** | **%** |
| --- | --- | --- |
| **Age (years)** | 42.4±12.1^a^ |  |
| **Diagnostic category** |  |  |
| Normal | 476 | 44.2 |
| Low grade CIN | 99 | 9.2 |
| High grade CIN | 301 | 28.0 |
| Cervical cancer | 181 | 16.8 |
| Metastasis | 19 | 1.8 |
| **FIGO stage** |  |  |
| I | 118 | 65.2 |
| II | 54 | 29.8 |
| IV | 9 | 5.0 |
| **Tumor grade**^b^ |  |  |
| Well+Moderate | 107 | 64.1 |
| Poor | 60 | 35.9 |
| **Cell type** |  |  |
| Squamous cell carcinoma | 148 | 81.8 |
| Others | 33 | 18.2 |
| **Tumor size** |  |  |
| < 4cm | 126 | 72.6 |
| ≥ 4cm | 49 | 27.4 |
| **LN metastasis**^c^ |  |  |
| Negative | 113 | 79.6 |
| Positive | 29 | 20.4 |
| **Chemoradiation**^d^ |  |  |
| Good response | 36 | 76.6 |
| Bad response | 11 | 23.4 |

CIN, cervical intraepithelial neoplasia; FIGO, International Federation of Gynecology and Obstetrics; LN metastasis, Lymph node metastasis; SCC, squamous cell carcinoma.

^a^mean±SD, ^b^calculated only 167 cases with available information of tumor grade, ^c^calculated only 142 cases with available information of examined lymph node, ^d^calculated only 47 cases with available information of chemoradiation response.
