# Supplementary material for: Synaptonemal Complex Protein 3 Is a Prognostic Marker in Cervical Cancer
Source: PLoS One. 2014 Jun 6;9(6):e98712. doi: 10.1371/journal.pone.0098712 (PMC4048308; doi:10.1371/journal.pone.0098712)
Supplement: Table S2 — Expression of SCP3 and pAKT in relation to clinicopathological characteristics in IHC analysis. (DOCX) [file pone.0098712.s006.docx]

**Table S2.** Expression of SCP3 and pAKT in relation to clinicopathological characteristics in IHC analysis

|  |  |  | **SCP3** |  |  |  | **pAKT** |  | |
| --- | --- | --- | --- | --- | --- | --- | --- | --- | --- |
|  | No. | % | Mean scores  (95% CI) | *p value* | No. | % | Mean scores  (95% CI) | | *p value* |
| **All study subjects** | 1029 | 100 | 4.62 (4.48-4.77) |  | 1010 | 100 | 4.59 (4.41-4.76) | |  |
| **Diagnostic category** |  |  |  | <0.001 |  |  |  | | <0.001 |
| Normal | 458 | 44.5 | 3.10 (2.96-3.24) |  | 446 | 44.2 | 2.84 (2.68-3.00) | |  |
| Low grade CIN | 97 | 9.4 | 3.77 (3.51-4.03) |  | 89 | 8.8 | 4.02 (3.54-4.50) | |  |
| High grade CIN | 280 | 27.3 | 5.71 (5.50-5.93) |  | 278 | 27.5 | 5.61 (5.30-5.92) | |  |
| Cancer | 176 | 17.1 | 7.00 (6.70-7.29) |  | 178 | 17.6 | 7.24 (6.86-7.61) | |  |
| Metastasis | 18 | 1.7 | 7.83 (6.94-8.72) |  | 19 | 1.9 | 8.47 (7.10-9.85) | |  |
| **FIGO stage** |  |  |  | 0.002 |  |  |  | | 0.472 |
| I | 116 | 65.9 | 6.63 (6.23-7.03) |  | 116 | 65.2 | 7.09 (6.63-7.54) | |  |
| II | 52 | 29.6 | 7.66 (7.30-8.01) |  | 53 | 29.8 | 7.53 (6.80-8.26) | |  |
| IV | 8 | 4.5 | 8.00 (7.05-8.94) |  | 9 | 5.0 | 7.78 (5.68-9.88) | |  |
| **Tumor grade** |  |  |  | <0.001 |  |  |  | | 0.036 |
| Well+Moderate | 105 | 64.8 | 6.37 (5.95-6.79) |  | 107 | 64.4 | 6.90 (6.42-7.39) | |  |
| Poor | 57 | 35.2 | 8.00 (7.69-8.32) |  | 59 | 35.6 | 7.83 (7.12-8.54) | |  |
| **Cell type** |  |  |  | 0.844 |  |  |  | | 0.093 |
| SCC | 144 | 81.8 | 7.01 (6.68-7.34) |  | 145 | 81.5 | 7.08 (6.68-7.49) | |  |
| Others | 32 | 18.2 | 6.93 (6.21-7.65) |  | 33 | 18.5 | 7.91 (6.90-8.92) | |  |
| **Tumor size** |  |  |  | 0.836 |  |  |  | | 0.766 |
| ≤ 4 cm | 124 | 70.5 | 6.98 (6.64-7.31) |  | 124 | 69.7 | 7.29 (6.85-7.73) | |  |
| > 4 cm | 52 | 29.5 | 7.04 (6.44-7.65) |  | 54 | 30.3 | 7.17 (6.42-7.91) | |  |
| **LN metastasis** |  |  |  | 0.054 |  |  |  | | 0.309 |
| No | 111 | 80.4 | 6.55 (6.15-6.96) |  | 111 | 79.3 | 7.12 (6.65-7.59) | |  |
| Yes | 27 | 19.6 | 7.42 (6.63-8.22) |  | 29 | 20.7 | 7.66 (6.65-8.66) | |  |
| Chemoradiation |  |  |  | 0.005 |  |  |  | | 0.329 |
| Good response | 35 | 79.5 | 5.98 (5.13-6.83) |  | 35 | 76.1 | 7.31 (6.39-8.24) | |  |
| Bad response | 9 | 20.5 | 7.77 (6.81-8.74) |  | 11 | 23.9 | 7.91 (7.04-8.78) | |  |

CIN, cervical intraepithelial neoplasia; FIGO, International Federation of Gynecology and Obstetrics; LN metastasis, Lymph node metastasis; SCC, squamous cell carcinoma.
